# A Rare Case of Chromoblastomycosis Resembling Keloid in an Indonesian Child

**DOI:** 10.7759/cureus.18490

**Published:** 2021-10-05

**Authors:** Fatima A Khairani, Ruri D Pamela

**Affiliations:** 1 Dermatology, Prabumulih Regional General Hospital, South Sumatera, Prabumulih, IDN; 2 Dermatology, Suyoto Hospital Centre of Rehabilitation, Ministry of Defense, Jakarta, IDN

**Keywords:** sclerotic cell, muriform cell, fungi, copper pennies, chromoblastomycosis

## Abstract

Chromoblastomycosis (CBM) is a rare chronic fungal infection caused by various dematiaceous fungi. This mycosis is mostly found in middle-aged males in tropical and subtropical countries. Only few cases of CBM in children have been reported. The diagnosis of CBM is often delayed due to the similarities with other dermatological diseases, such as cutaneous tuberculosis, mycetoma, leprosy, viral warts, psoriasis vulgaris, or malignancies.

We report a case of an 11-year-old healthy boy having CBM. On his left knee, there were large erythematous plaques and tumors with scaly surfaces, some lesions appeared to be cauliflower-like. The patient denied pain and pruritus. The preliminary diagnosis was keloid; however, histopathological findings led to the final diagnosis, which was established as CBM. The patient was treated with oral itraconazole 100 mg daily. His lesions partially resolved within one month of treatment.

Although uncommon in children, the differential diagnosis of CBM must be considered in any suspicious lesion(s). Itraconazole 100 mg daily gave a good response in children with CBM. Accurate diagnosis and early treatment are needed to achieve successful management of CBM in children.

## Introduction

Chromoblastomycosis (CBM) is a chronic and progressive infection of the skin and subcutaneous tissue caused by various dematiaceous fungi [1-3]. This mycosis generally occurs in middle-aged males [2], and is rarely found in children and adolescents [3-5]. Most cases were reported from humid tropical and subtropical areas of Asia, Africa, and America [3, 6-7].

The clinical manifestations of CBM may vary; it might present as nodules, tumors, plaques, warty, and scarring lesions [2, 4, 7]. The lesions are often seen in the lower limbs, and the face and upper limb are seldom affected [6, 8]. Sometimes, the diagnosis of CBM is delayed because of clinical simulation of other dermatological diseases. The differential diagnoses of CBM are cutaneous tuberculosis, leprosy, leishmaniasis, sporotrichosis, mycetoma, viral warts, psoriasis vulgaris, sarcoidosis, lupus erythematosus, or malignancies [6-7, 9]. The lesions of CBM grow slowly, and sometimes leaving sclerotic plaques or keloid [3, 10].

We reported a case of CBM of the left knee in an 11-year-old boy from South Sumatra, Indonesia, who was earlier misdiagnosed as keloid. This report discusses the clinical manifestations, therapy, and outcome of the disease.

## Case presentation

An 11-year-old boy from South Sumatera, Indonesia, presented with slowly growing, scaly erythematous plaques on his left knee. The lesions were neither associated with itching nor pain. His father said that his son developed a small red plaque on the left knee five years ago, which gradually increased in size. The patient reported frequent bicycle riding and previous history of knee injuries, such as mild abrasions. There were no similar lesions among family members. The boy denied having many outdoor activities associated with soil or plants. There was no history of allergy.

**Figure 1:**
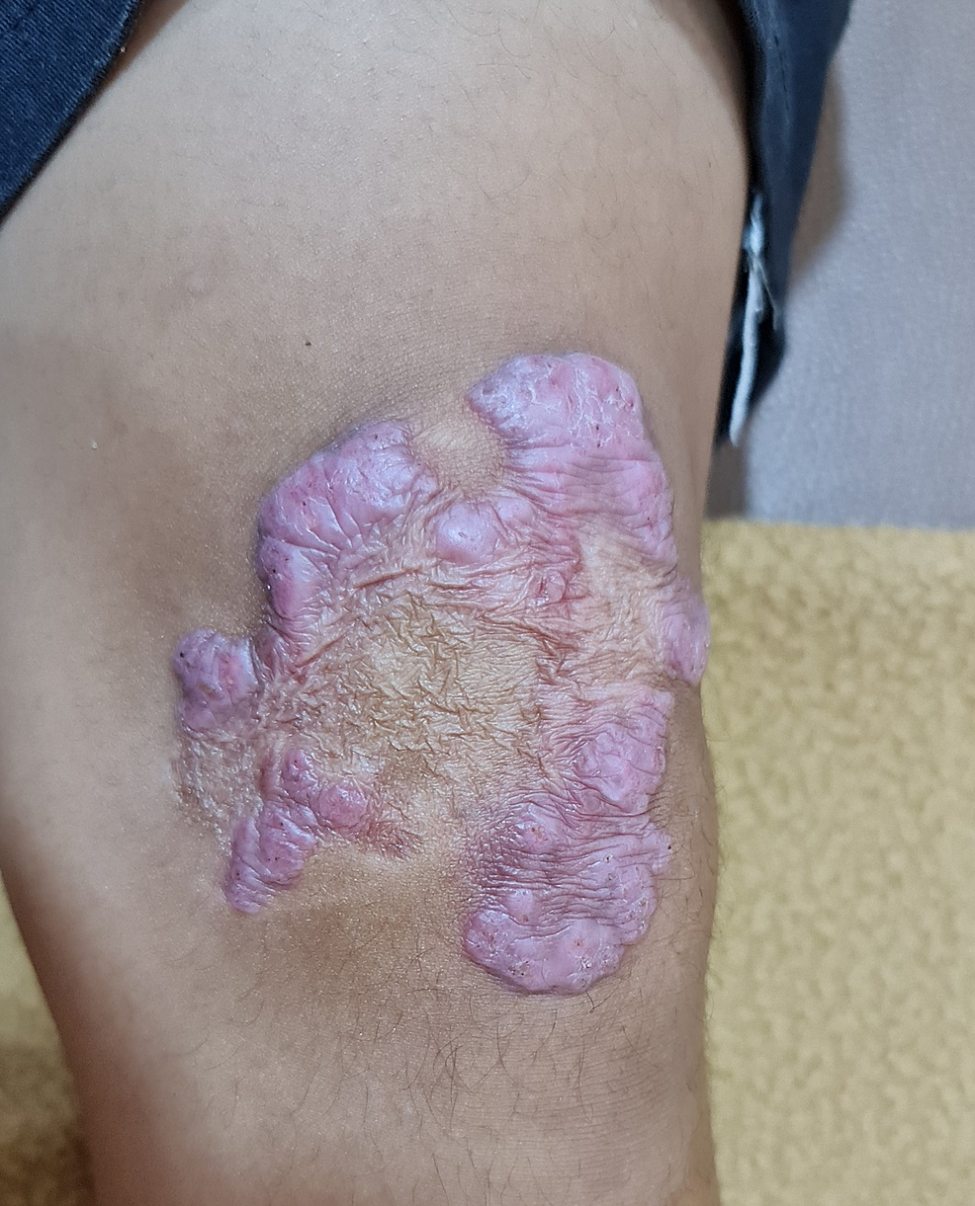
Clinical manifestations at admission. Large erythematous plaques and tumors resembling cauliflower-like masses on left knee

On physical examination, the general condition and vital signs of the boy were within normal limits. He was moderately built and well nourished. On his left knee, there were large erythematous plaques and tumors with scaly surfaces, some lesions resembled cauliflower-like masses (Figure 1).

The patient was initially diagnosed as keloid with psoriasis vulgaris as a differential diagnosis because some of the lesions consist of erythematous plaques with scales. Due to the unusual nature of the suspected keloid lesion, skin biopsy samples were taken and subjected to histopathological examination.

**Figure 2:**
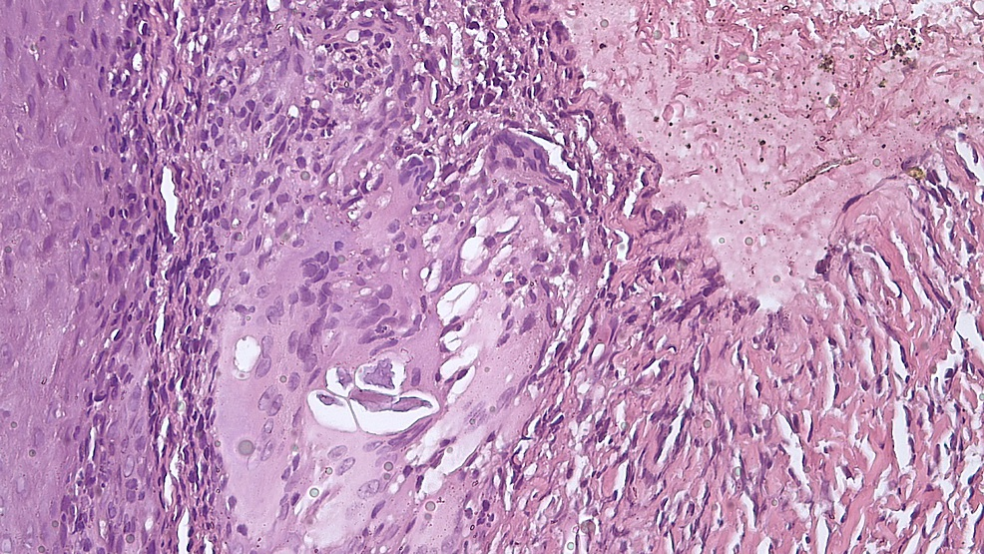
Histopathological examination. Lymphocytic and neutrophilic infiltrates with dermal epitheloid granulomas

**Figure 3:**
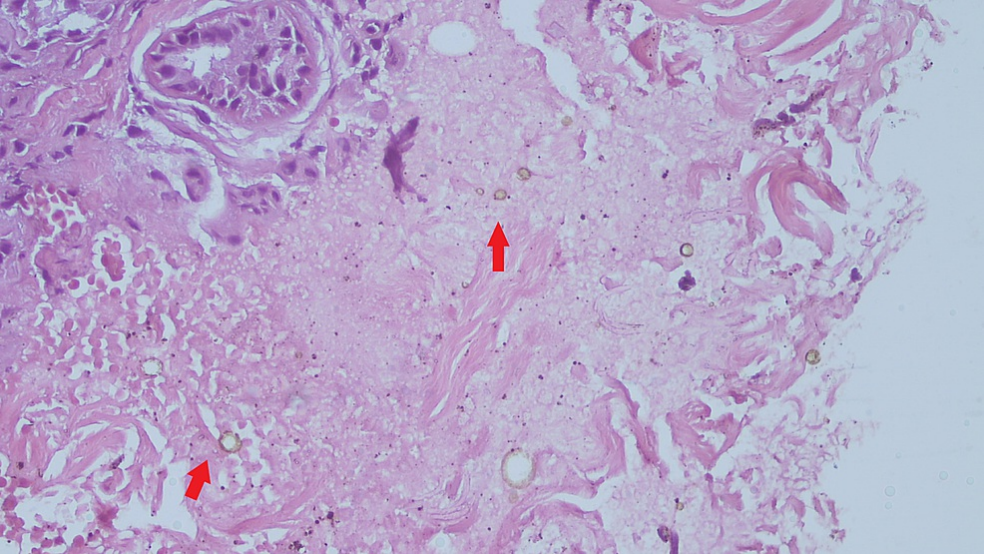
Muriform cells or sclerotic cells. Muriform cells or sclerotic cells (copper-pennies) pathognomonic for chromoblastomycosis found within the tissue

The histopathological examination showed lymphocytic and neutrophilic infiltrates with dermal epitheloid granulomas. Multiple sclerotic cells (‘copper-pennies’) that were brown-colored with a thick wall were found within the tissue (Figures 2-3).

**Figure 4:**
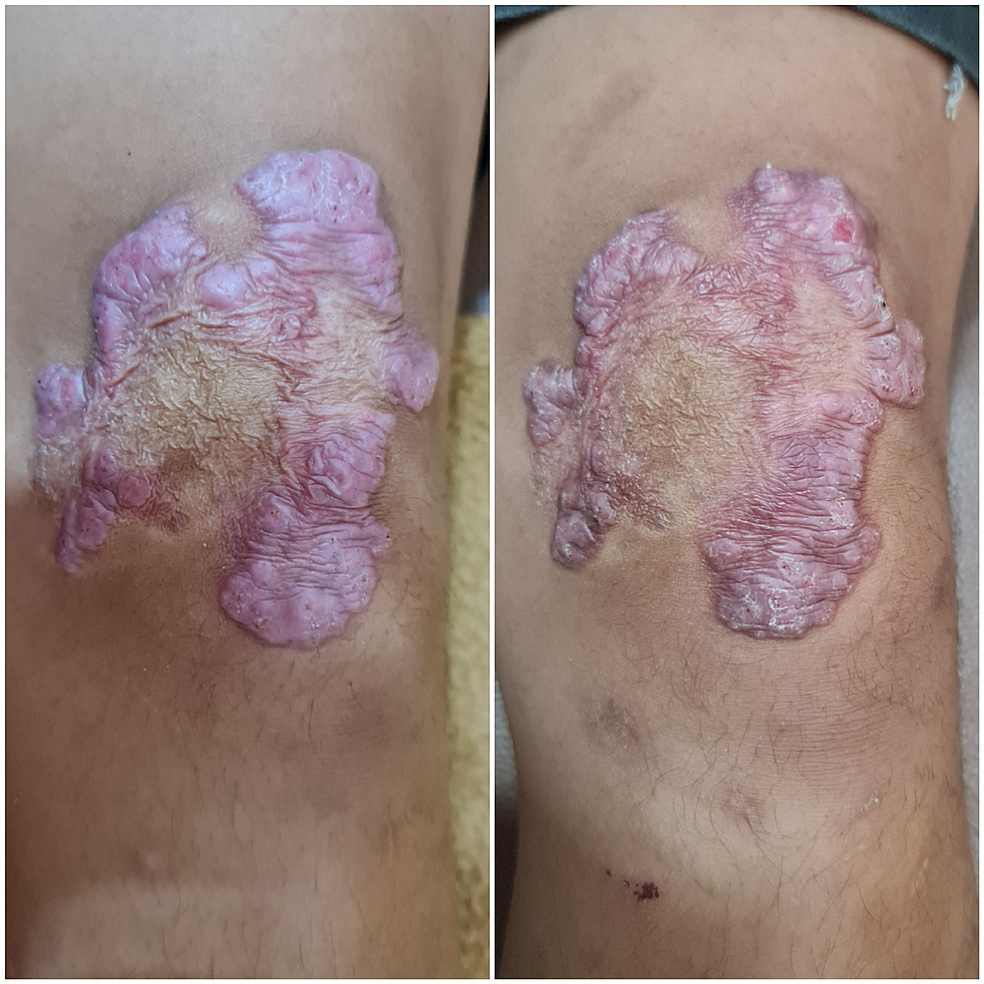
Improvement in patient. Lesions became thinner and smaller after one month of therapy with itraconazole 100 mg a day (right figure)

The diagnosis of CBM was made in this patient. Oral itraconazole 100 mg a day was started, and as secondary cutaneous bacterial infection was suspected, cefixime 100 mg twice a day for seven days was also given to this patient. Improvement was seen within one month of therapy (Figure 4). The patient and his father were satisfied, and until this report was made, the patient is still following with us on itraconazole to complete a total of 12 months of therapy.

## Discussion

Chromoblastomycosis was first described by Pedroso and Gomes in 1920 in Brazil. It was previously known as ‘verrucous dermatitis of infectious origin’ [3]. The most common causes of CBM are *Fonsecaea pedrosoi* and *Cladophialophora carrionii* [7-8], which usually infect human tissues after traumatic implantation through the skin [3]. CBM can affect individuals belonging to different age groups, but has been infrequently reported among children and adolescents [3, 7]. Perez-Blanco et al. reviewed cases of CBM from 1992 to 2004 in Falcon State, Venezuela, and observed 22 cases in children and adolescents [5]. Based on our best knowledge, there is no prior report of CBM case in children from Indonesia.

Several reasons explain the lack of reports in pediatric group. Initial symptoms may not be significant enough for a child to complain and seek medical or professional help [5]. Meanwhile, the incubation period of CBM is not well defined, and the disease progresses slowly over months or years [7]. Therefore, an adult diagnosed with CBM might have been infected initially in childhood or adolescence. [5]. As often diagnosed in adults, CBM in children can also be misdiagnosed due to its similarity with other dermatological or infectious disorders, such as cutaneous tuberculosis, sporotrichosis, mycetoma, leprosy, leshmaniasis, psoriasis, and malignancies [4, 6-7, 9].

Infection of CBM is acquired from fungal elements which are transmitted from the environment through the skin, usually following trauma on the extremities [3-4, 6]. CBM is due to filamentous fungi of the Dematiaceae family [7], with the genera Fonsecaea, Phialophora, and Cladophialophora as the most common cause [3]. These fungi are found as saprophytes in soil and plants, such as rotting wood [3-4]. The lesions of CBM usually affect the lower limbs [8]. However, cases of CBM also have been reported in upper limbs [4], buttocks, and trunk [5]. In our report, the patient reported a history of bike rides and thereby having had skin abrasions or scrapes of his knee, which could have led to the introduction of the fungi into his cutaneous tissue. He denied having many activities associated with soil or plants. Nevertheless, we could not identify the definite factors of the infection in our patient.

There are several factors in the pathogenesis of CBM such as fungus virulence, host genetic susceptibility, and repeated exposure to the etiological agents [6]. Adult age, male sex, rural worker, low socioeconomic groups, poor nutrition, and poor personal hygiene are the known risk factors of CBM [6, 11]. Santos et al. analyzed 191 cases of CBM in Brazil, and found only two patients had immunosuppressive diseases on diagnosis of CBM, which was kidney transplantation and cutaneous lupus erythematosus [12]. Meanwhile, Srinivas et al. reported a case of CBM in a 12-year-old immunocompetent male child [13]. Similar to our case, our patient was an immunocompetent child, well-nourished, without any significant comorbidities. 

Chromoblastomycosis has an insidious course [3, 7, 10] without clear definition of its incubation period. The lesions expand in size, rather than invade deeply to the muscle or bone [7]. It may begin as erythematous papule which develop into plaques [10], nodules, or verrucous lesions. In the coming years, lesions may progress into tumoral, cauliflower-like masses. Lesions can also spread centripetally, thus resulting in scarring on the central areas of lesions [3]. Afterwards, the lesions may become sclerotic plaques or keloids [10]. Carrión in 1950 classified the cutaneous manifestations of CBM into five types: nodular, plaque, tumoral (cauliflower-like), cicatricial, and verrucous lesions [6-8]. However, two or more types of lesions can be present in a same patient [6, 8]. CBM is usually asymptomatic, but itching and pain can be found in advanced cases [6].

Investigations that aid in establishing a diagnosis include mycological exam (direct examination and culture) and/or histopathology [6]. The direct examination using potassium hydroxide (KOH) mounts shows muriform cells, which are viewed as brown colored, rounded, thick-walled structures with a single or double septum [3, 7]. These cells, also known as sclerotic cells or ‘copper-pennies’, are pathognomonic for CBM regardless of the causative fungi [3]. From histopathological findings, there are marked epithelial hyperplasia, chronic granulomatous infiltrates with multinucleated giant cells, epithelioid cells, histiocytes and lymphocytes, also presence of muriform cells [3-4]. Culture is performed on Sabouraud dextrose agar [7], showing slow-growing dark fungi [3] which start off as deep green progressing to black in color [6-7]. However, it may be inadequate to determine the exact causative agent because of the poor morphological differentiation. Polymerase chain reaction (PCR) assays have been chosen for detection of the etiological agents, such as Fonsecaea species and *C. carrionii* [3].

The direct examination with KOH was not done in this patient because initially we considered the differential diagnosis were keloid and psoriasis vulgaris. Culture and PCR assays were not completed in this patient, as these facilities are not currently available in our practice setting. Based on the histopathological examination, we concluded that the diagnosis in this patient was CBM. However, the exact etiological agent was not revealed in this patient, and this is a limitation of our case report.

There is no gold standard treatment for CBM [6, 8]. The management of CBM is difficult and challenging [8, 10, 14], although many treatment options can be considered. The therapy of CBM involves long antifungal chemotherapy, sometimes combined with physical methods, such as surgery, cryotherapy, and thermotherapy [2-3]. The outcome of treatment depends on the severity of the lesions, the etiological agent [2-3, 10], and the presence of complications. Skin edema and fibrosis in CBM may reduce the bioavailability of the antifungal drugs in the tissue [2-3]. *Fonsecaea pedrosoi* is less sensitive to antifungal therapy than *C. carrionii* or *Phialophora verrucosa* [3]. The cure criteria in CBM consist of clinical, mycological, and histopathological criteria [8]. There should be complete recovery in all lesions, leaving scar [2] without any symptoms such as itching or pain. The mycological examination is defined by no fungal elements in direct examination and culture [2, 8]. The histopathological examination would reveal chronic inflammation replacing the active granulomatous infiltration, with no muriform cells [8].

Itraconazole and terbinafine are the most effective antifungal drugs for CBM [3]. The drugs should be given for a minimum of 6-12 months [3, 8]. In refractory cases, combination antifungal therapy may be attempted [8]. Itraconazole is a broad-spectrum fungistatic triazole, acts through the suppression of ianosterol to ergosterol by blocking the 14-alpha-demetilase. It is a safe choice for long duration treatment in CBM [8]. Other antifungal drugs such as ketoconazole and fluconazole are not recommended for CBM therapy. Ketoconazole may induce toxicity in high doses, and fluconazole may not be effective for dematiaceous fungi [3].

There are several reports about the use of itraconazole for CBM in children and adolescents. Pradeepkumar et al. reported a nine-year-old Indian child with CBM who was treated with itraconazole 100 mg daily and terbinafine 250 mg daily. The mycological examination was negative two months after treatment [4]. Pérez-Blanco et al. reviewed 22 cases of CBM in children and adolescents in an endemic area of the Falcon State, Venezuela. Two children were given itraconazole 100 mg daily for one month and showed a good response to therapy [5]. Srinivas et al. reported one case of CBM in a 12-year-old immunocompetent male child who was successfully treated with itraconazole 200 mg daily for one year [13].

## Conclusions

Chromoblastomycosis is a rare pediatric infectious disease. However, the differential diagnosis of CBM must be thought in patients with long standing and unusual clinical manifestations. Our report highlights the understanding of this mycoses; therefore, prevents the delayed treatment and improves the quality of life in patients, especially in children.
